# Epidemiology of measles in the metropolitan setting, Addis Ababa, Ethiopia, 2005–2014: a retrospective descriptive surveillance data analysis

**DOI:** 10.1186/s12879-018-3305-4

**Published:** 2018-08-14

**Authors:** Munira Nasser Hassen, Abyot Bekele Woyessa, Mekonen Getahun, Berhane Beyene, Lucy Buluanger, Ayesheshem Ademe, Alemayehu Bekele, Adamu Addissie, Amha Kebede, Daddi Jima

**Affiliations:** 1 Addis Ababa Health Bureau, Addis Ababa, Ethiopia; 2 Center for Public Health Emergency Management, Ethiopian Public Health Institute, PO BOX 1242, Addis Ababa, Ethiopia; 3 CDC, Addis Ababa, Ethiopia; 4 WHO, Addis Ababa, Ethiopia; 5 Addis Ababa University, Addis Ababa, Ethiopia; 6 Ethiopian Public Health Association, Addis Ababa, Ethiopia

**Keywords:** Measles, Surveillance, Vaccination, Outbreak, Addis Ababa, Ethiopia

## Abstract

**Background:**

Measles is a highly infectious and serious respiratory viral disease which caused by a virus. It is a significant cause of illness and death worldwide. This data analysis was conducted to describe the trend and determine the reporting rate of measles cases in Addis Ababa to make recommendation for the government of the city to strengthening measles control interventions.

**Methods:**

We obtained and extracted ten years (2005–2014) Addis Ababa city’s measles surveillance data from national database. We carried out retrospective descriptive data analysis by time, place and person variables. We calculated cumulative and specific reporting rates by dividing measles cases (lab confirmed, epidemiologically linked and compatible cases) to respective population and multiplying by 100,000. We divided average of ten years measles cases to midyear population and multiplied by 100,000 to calculate annualized reporting rate. We analyzed non-measles febrile rash rate by dividing laboratory negative cases to total population and multiplying by 100,000.

**Results:**

A total of 4203 suspected measles cases were identified. Among them 1154 (27.5%) were laboratory confirmed, 512 (12.2%) were clinically compatible, 52 (1.2%) were epidemiologically linked cases and the rest 2485 (59.1%) were IgM negative for measles which makes total measles cases 1718 (40.9%). Median age was 5 years with 2–18 years interquartile-range. The annualized measles reporting rate was 5.9, which was 40.2 among > 1 year, 11.5 among 1–4 years, 6.0 among 5–14 years, 4.1 among 15–44 years and 0.01 among ≥ 45 years per 100,000 population. Among the total measles cases; 380 (22%) were received at least one dose of measles containing vaccine (MCV) while 415 (24%) cases were not vaccinated and the vaccination status of 923 (54%) cases were not known.

**Conclusion:**

Our analysis revealed that the reporting rate was higher among young children than older age group. Among all the patients 22% were received at least one dose of measles vaccine whereas 13% were not vaccinated against measles antigen. Routine immunization should be strengthened to reach all children through well monitored vaccine cold chain management.

## Background

Measles is a vaccine preventable, highly infectious and serious respiratory disease caused by measles virus [1–3]. It is illustrated by a prodrome of fever (as high as 105 °F) and malaise, cough, coryza, and conjunctivitis, followed by a maculopapular rash [4]. The rash typically appears 14 days after exposure to the virus and spreads from head to trunk to lower extremities [5]. It is highly communicable disease that is a significant cause of illness and death worldwide [6, 7]. It is transmitted from person to person through air, droplet or by direct contact with the nasal and throat secretions of infected persons [8]. After exposure to the virus, up to 90% of susceptible persons develop measles infection [9].

Measles vaccination has markedly reduced the incidence of measles virus infection and is one of the most successful global public health interventions; it prevents millions of deaths annually, primarily among infants and young children [10]. In Africa before the introduction of measles vaccination, measles was mostly a disease affecting young children, and more than 1 million cases were reported annually [11]. Number of measles cases decreased by 81%, from 520,102 cases in 2000 to 98,621 cases in 2015. Measles incidence rate decreased by 85%, from 841 cases in 2000 to 100 cases in 2015 per million population in African region with estimated 85% mortality reduction from 2000 to 2015 [12].

Ethiopia adopted the African regional accelerated measles control strategies to reduce measles mortality in 2002. Routine measles vaccination is provided to infants at 9 months of age and a second opportunity for measles vaccination through supplementary immunization activities (SIAs), targeting children aged 6 months to14 years [13, 14]. Routine immunization services are provided in most of the health facilities and in outreach services for communities residing beyond 5 km from the static health facilities by trained health workers. Currently, all public health facilities providing immunization services. Some private hospitals in Addis Ababa also providing immunization activities. The program is funded primarily by partners and Government and provided free of charge in public health facilities and facilities supported by Non-Governmental Organizations (NGOs). The partners largely channel their funds through UNICEF and WHO [15].

In Ethiopia, measles is one of nationally immediately notifiable diseases [16] that all health facilities (private and public) are expected to report to the next level public health agency using the aforementioned case definitions. Clinicians (internist, general practitioners, nurses, health officers) reported suspected measles cases with blood serum samples using case-based reporting form to Ethiopian Public Health Institute for laboratory analysis [13]. Ethiopian Public Health institute is mandated by councils of ministers regulation to conduct surveillance and laboratory investigations on epidemic prone diseases including measles [13, 17].

We conducted a retrospective data analysis to describe trend and reporting rate of measles cases by time and age group and assess the vaccination status of measles cases in a metropolitan city, Addis Ababa. It is also to identify measles surveillance and immunization gaps to make possible recommendations for the city government to strengthen measles detection, control and prevention interventions.

## Methods

**Fig. 1:**
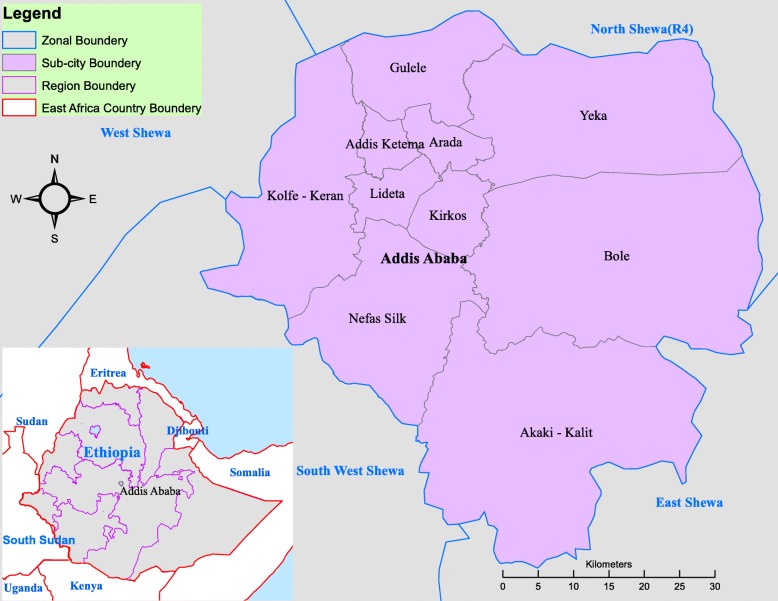
Map showing Addis Ababa Sub cities (Source: Shapefile from Ethiopian Karta Agency)

We performed retrospective descriptive data analysis on measles routine surveillance data reported from 2005 to 2014 from Addis Ababa City. Addis Ababa is a capital of Ethiopia which divided in to ten sub-cities. The city is enclosed by Oromia regional state zones’ in all directions (Fig. 1). As per the 2007 census projection the total population of Addis Ababa is estimated to 3,167,035 for 2014.

We obtained and extracted 10 years (2005–2014) Addis Ababa’s measles surveillance data from Ethiopian Public Health Institute (EPHI) national surveillance database. We defined suspected measles cases as any person with fever and maculopapular (nonvesicular) generalized rash and cough, coryza or conjunctivitis (red eyes) which are diagnosed by clinician OR any person in whom a clinician suspects measles: We also further classified the suspected measles cases collected through surveillance into four categories as per the national and WHO guidelines. 1) Laboratory confirmed measles cases: Is a suspected measles cases which has laboratory results indicating measles IgM positive. 2) Epidemiologically linked measles case: A suspected measles case that has not had a blood specimen taken for serologic confirmation and is linked (in place, person and time) to a laboratory confirmed case; i.e., living in the same or in an adjacent district with a laboratory confirmed case where there is a likelihood of transmission; onset of rash of the two cases being within 30 days of each other. 3) Compatible case is a suspected case which has not been adequately investigated. 4) Discarded measles case: is a suspected measles case which, upon adequate investigation that includes a blood specimen collected in the appropriate time frame, lacks serologic evidence of a measles virus infection or measles IgM negative cases [13, 18, 19]. We considered the sum of laboratory confirmed, clinically compatible and epidemiologically confirmed cases as measles. Only a person received at least one dose of measles containing vaccine (MCV) is considered as vaccinated against measles.

We performed data cleaning and checked for duplication by patient name, sex, age, residence area, date of onset, date seen at health facility and date sample collection. We used the 2007 population census [20] projection as denominator in calculating cumulative and age, sex and area specific measles reporting rates. We divided the number of measles cases to population and multiplied by 100,000 to calculate cumulative reporting rate. We divided age, year, and area specific measles cases to respective population and multiplied by 100,000 to calculate specific reporting rates. We also divided average of ten years measles cases to midyear population and multiplied by 100,000 to calculate annualized reporting rate. We also determined non-measles febrile rash case rate by dividing discarded or laboratory negative suspected measles cases to population and multiplying by 100,000. We used Arc Geographic Information System (GIS) version 10.2 to indicate study area.

## Results

From 2005 to 2014, a total of 4203 suspected measles cases were reported from ten sub-cities of Addis Ababa. Among the total suspected measles cases; 1154 (27.5%) were laboratory confirmed measles cases, 512 (12.2%) were clinically compatible measles cases, 52 (1.2%) were epidemiologically linked measles cases and the rest 2485 (59.1%) were measles IgM negative cases. Hence, total measles cases (laboratory confirmed, clinically compatible case and epidemiologically linked measles cases) were 1718 (40.9%). Among 1787 measles cases; 1442 (84%) were outpatient visits while the rest 276 (16%) were inpatient with two deaths attributed to measles which makes CFR 0.12%. Median age for measles cases was 5 years and interquartile range was 2–18 years. Of the total measles cases 274 (16%) were under 1 year, 405 (24%) were 1–4 year, 525 (31%) were 5–14 year, 513 (30%) were 15–44 and 1 (0.1%) were above 44 years. The non-measles febrile rash case rate increased from 3.4 in 2009 to 11.1 in 2014 per 100,000 population having an average of 8.6 annualized non-measles febrile rash case rate in ten years (Table 1).

**Table 1:** Category of measles, reporting rate and non-measles febrile rash illness rate per 100,000 population by year, Addis Ababa, 2005–2014

Year	Suspected Cases	Confirmed by lab	Epi linked	Compatible	Discarded	Total Measles	Non-Measles Rash case Rate	Median age	Age IQR
2005	148	6	0	4	138	10	5.2	5	4–9
2006	422	141	17	8	256	166	9.6	6	2–17
2007	188	65	0	1	122	66	4.5	6	2–15
2008	228	43	4	5	176	52	6.3	3	1–7
2009	241	43	22	80	96	145	3.4	6	3–9
2010	749	238	0	283	228	521	7.8	6	1–15
2011	315	77	8	40	190	125	6.4	7	1–21
2012	553	36	0	4	513	40	16.9	7	2–17
2013	653	237	0	14	402	251	13.0	12	4–20
2014	706	268	1	73	364	342	11.5	10	3–16
Total	4203	1154	52	512	2485	1718	8.6	5.0	2–18

The annualized measles reporting rate was 5.9 per 100,000 population. The annualized reporting rate was 40.2 among less than one-year age, followed by 11.5 among children from 1 to 4, 6.0 among 4–14 years, 4.1 among 15–44 and 0.3 among 45 years and above per 100,000 population. Relatively the reporting rate was high in Akaki Kality sub-city followed by Kolfe Keranio sub-city (7.4 and 7.3 measles cases per 100,000 population respectively). The reporting rate was decreasing as the age group increasing in all sub-cities (Table 2).

**Table 2:** Annualized measles reporting rate per 100,000 population by sub city and age group, Addis Ababa, 2005–2014

Region	< 1	1–4	5–14	15–44	44+	Annualized
Addis Ketema	44.1	10.6	5.0	2.4	–	4.9
Akaki Kaliti	17.7	9.0	10.5	6.3	–	7.4
Arada	53.0	8.8	7.6	3.1	–	6.0
Bole	40.3	12.3	4.7	5.6	–	6.3
Gullele	48.1	16.2	6.8	4.0	–	6.9
Kirkos	52.7	11.6	5.1	3.0	–	5.5
Kolfe Keranio	51.5	12.8	6.4	5.6	–	7.1
Lideta	47.8	11.1	5.4	2.8	–	5.4
Nifas Silk Lafto	25.4	11.0	5.6	4.0	0.2	5.4
Yeka	22.0	9.4	4.5	3.5	–	4.5
Annualized	40.2	11.5	6.0	4.1	0.0	5.9

**Fig. 2:**
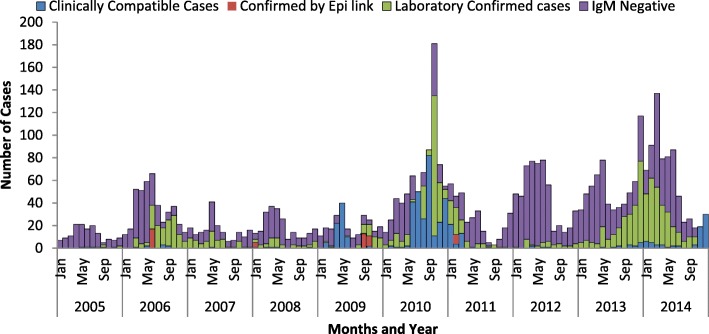
Trends of Measles Cases by Classification by Months, Addis Ababa, 2005–2014

**Fig. 3:**
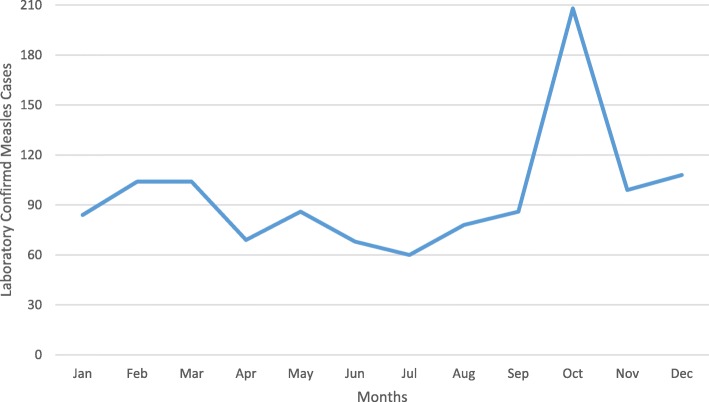
Trends of laboratory confirmed measles cases by month, Addis Ababa, 2005–2014

**Fig. 4:**
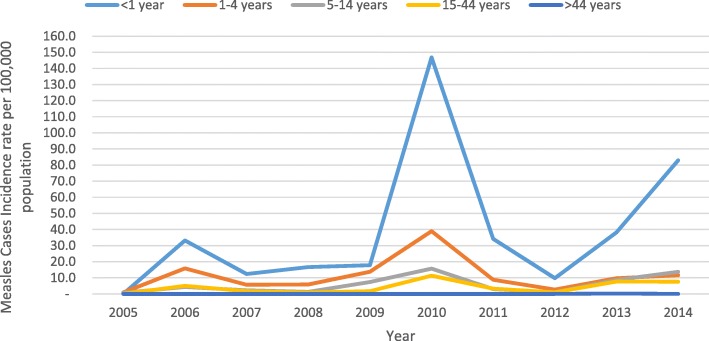
Trends of measles reporting rate per 100,000 population over years by age group, Addis Ababa, 2005–2014

Annual measles reporting rate was different from year to year. Relatively, reporting rate was high in 2010 followed in 2014, 2013 and 2006 which is 18, 11, 8 and 6 cases per 100,000 population respectively. The lowest proportion of laboratory confirmed measles cases were reported in July while the highest proportion of the cases were reported in October and December (Figs. 2 and 3). The annual reporting rate was relatively high among under one-year age groups followed among 1–4 years across ten years ((Table 3, Fig. 4).

**Table 3:** Measles reporting rate per 100,000 population over years by age group, Addis Ababa, 2005–2014

Year	Age group specific measles incidence rate					Annual incidence arte
	< 1 year	1–4 years	5–14 years	15–44 years	> 44 years	
2005	–	0.9	0.8	0.1	–	0.4
2006	33.2	15.8	4.3	5.0	–	6.2
2007	12.4	5.7	2.3	1.7	–	2.4
2008	16.7	5.8	1.3	0.8	–	1.9
2009	17.8	13.7	7.4	1.7	–	5.1
2010	146.8	39.0	15.7	11.3	–	17.9
2011	34.2	8.8	3.0	3.3	–	4.2
2012	9.8	2.7	1.1	1.0	–	1.3
2013	38.2	9.7	8.7	7.7	0.3	8.1
2014	83.0	11.6	13.7	7.6	–	10.8
Annualized	40.2	11.5	6.0	4.1	0.03	5.9

Among the 1718 measles cases; 380 (22%) were received at least one dose of measles containing vaccine (MCV) while 415 (24%) were not vaccinated and the rest 923 (54%) vaccination status were not known. From the total 1154 laboratory confirmed measles cases; 164 (14%) were took at least one doses of measles vaccine, 280 (24%) were not vaccinated and the vaccination status of 710 (62%) were not known (Table 4).

**Table 4:** Measles cases vaccination status (number/percent) versus final case classification, Addis Ababa, 2005–2014

Vaccination Status	Confirmed by lab	Epi link cases	Compatible cases	Total Measles	IgM Negative	Total
One dose	135 (12)	4 (8)	172 (34)	311 (18)	840 (34)	1151 (27)
Two doses	16 (1)	0 (0)	25 (5)	41 (2)	356 (14)	397 (9)
> 2 doses	13 (1)	0 (0)	15 (3)	28 (2)	83 (3)	111 (3)
At least one dose	164 (14)	4 (8)	212 (41)	380 (22)	1279 (51)	1659 (39)
Unknown	710 (62)	12 (23)	201 (39)	923 (54)	1066 (43)	1989 (47)
Unvaccinated	280 (24)	36 (69)	99 (19)	415 (24)	140 (6)	555 (13)
Total	1154 (100)	52 (100)	512 (100)	1718 (100)	2485 (100)	4203 (100)

## Discussion

We found that measles cases and reporting rates have been increased in Addis Ababa city from 2005 to 2014. The reporting rate was increased to topmost in 2010 followed by in 2014 and 2013. Corresponding to the increment of the reporting rate, non-measles rash illness rate was also increased by two folds. The increment of the non-measles febrile rash illness rate is an indicator for the sensitivity of the surveillance system. We believe that the increment of the cases and reporting rate of measles observed might be attributed to the sensitivity of surveillance system. The reporting or non-measles febrile rash illness rate was found to be greater than minimum WHO requirement which ≥2 per 100,000 per year [18, 19] which indicates that the measles surveillance system is sensitive in the capital. In addition, prolonged outbreak between 2013 and 2014 significantly increases measles reporting rate (Fig. 2). We also believe that increment of the reporting rate was might be also attributed to unvaccinated children over years. We have also observed that, reporting rate of measles was relatively low in July and August and start increasing from September over years. This is coexistence with school closure in July and August and reopening in September which might be contributing risk factors. Evidence from other study demonstrated a clear peak in transmission of measles which is coinciding with the start of the school calendar and decrease during vacation [21].

The study revealed that, the CFR is lesser as compared with other studies in Africa [22, 23]. The comprehensive community based study by WHO indicated that the CFR in Sudan, Ethiopia, Kenya and Tanzania ranged from 3 to 4% [24] which is much higher. The lower the case fatality rate in this finding might be attributed with the improvement of early case detection, clinical treatment and access to health care deliveries in Addis Ababa which needs further investigation.

The analysis showed that, all age groups were affected having median age 5 and Interquartile age range 2–18 years. The study conducted in Malawi on measles outbreak indicates that Median age of case-patients was 7 years (Interquartile range 1–16) which supported our finding [25]. Our finding further revealed that the reporting rate was high in lower age groups. Infants < 1 year of age were mostly affected (40/100,000) followed by children 1–4 years (11/100,000), 5–14 years (6/100,000), 15–44 Years (4/100,000) and 45+ years (0.3/100,000). The previous study in Addis Ababa indicates that the Seronegative prevalence decreased from 66% in infants less than 9 months of age to 20.4% in 9–59 month old, 4.9% in 5–14 years old, and 0.7% in adults (15–49 years) which supported our finding [26]. We observed that the trends in measles reporting rate in different age groups over years was follow similar trend. We did not observed shift over time in age groups. In each year infants under five were mostly affected (Fig. 3).

In our analysis, we found that 24% of the measles cases were not received measles vaccine while the vaccination status of significant portion, 54%, of the cases were not known. Furthermore, from the laboratory confirmed measles cases again 24% were not vaccinated and the vaccination status of 62% were not known (Table 4). This suggests that, there is some gaps in routine vaccination coverage. Even in the areas where the vaccination coverage at 9th month is 100%, the effectiveness of measles vaccine is only 85% with single dose [27, 28]. In Ethiopia 1st dose measles vaccine has given at the age of 9 months [13] whereas there is no 2nd dose routine vaccination schedule. The second opportunity is only through supplementary immunization activities. In this finding, from all laboratory confirmed cases, only 2% received two and more disease of measles vaccine.

We believe that, there are sufficient health facilities with better health care deliveries including vaccination services are available in Addis Ababa as compared with other areas of the country. However, the increment of the reporting rate and occurrences of measles outbreak in Addis Ababa city indicates that the vaccination activities and other measles prevention and control interventions has limitation in the capital.

This study on retrospective measles surveillance data has some limitations. As we used secondary surveillance data, we could not have mentioned how vaccination status of cases were obtained. Hence, vaccination status of the cases was not validated. The number of measles cases and its epi-classification used in this study was based on available surveillance data which might be less representative because of lack of sensitivity of measles surveillance. Contributing risk factors for not vaccinating children in Addis Ababa were also not determined as risk determinants were not available in the data. Vaccine cold chain quality was also not accessed as we used secondary surveillance data.

## Conclusions

In summary, measles infection remains a problem to human especially to children including in the area where health care service is accessible. Our analysis revealed that the measles reporting rate was higher among young children than older age group in the capital of Ethiopia, Addis Ababa. Among all the patients associated with measles, 22% were received at least one dose of measles vaccine, whereas 22% were not vaccinated against measles antigen and the vaccination status of significant patients were not known. Routine immunization program needs to be evaluated and should be strengthened to reach all children through well monitored vaccine cold chain management and improve data quality to avoid unknown vaccination status.

## Abbreviations

CFR: Case fatality rate
EPHI: Ethiopian Public Health Institute
GIS: Geographic information system
IQR: Interquartile range
MCV: Measles-containing vaccines
NGO: Non-Governmental Organization
UNICEF: United Nations International Children Emergency Fund
WHO: World Health Organization

## References

[1] Gastanaduy Paul A, Redd Susan B, Clemmons Nakia S, Lee Adria D, Hickman Carole J, Rota Paul A, Patel M (2017). Chapter 7: Measles. CDC Surveill Man.

[2] Coughlin MM, Beck AS, Bankamp B, Rota PA (2017). Perspective on global measles epidemiology and control and the role of novel vaccination strategies. Viruses.

[3] Public Health Agency of Canada (2013). Guidelines for the Prevention and Control of Measles Outbreaks in Canada. Vol. 39, Canada Communicable Disease Report.

[4] Measles M, Region E, Rubella R, Mumps M (2012). Vaccine-preventable diseases: Signs , symptoms & complications.

[5] Kutty P, Rota J, Bellini W, Redd SB, Albert Barskey GW (2013). Measles. VPD Surveillance Manual. 6th ed.

[6] Katz SL, Gellin BG (1994). Measles vaccine: do we need new vaccines or new programs?. Science (80-).

[7] Allam MF (2009). Measles Vaccination. J Prev Med Hyg.

[8] World Health Organization. WHO Guidelines for Epidemic Preparedness and Response to Measles Outbreaks. Response. 1999;WHO/CDS/CS(May). Available from: http://www.who.int/emc.

[9] Centers for Disease Control and Prevention (2013). Prevention of measles, rubella, congenital rubella syndrome, and mumps, 2013: summary recommendations of the Advisory Committee on Immunization Practices (ACIP). Mmwr.

[10] William J, Moss DEG (2012). Measles Lancet.

[11] Goodson JL, Masresha BG, Wannemuehler K, Uzicanin A, Cochi S (2011). Changing epidemiology of measles in Africa. J Infect Dis.

[12] Patel MK, Gacic-dobo M, Strebel PM, Dabbagh A, Mulders MN (2016). Progress Toward Regional Measles Elimination. Worldwide, 2000–2015.

[13] Ethiopian Public Health Institute (2012). Guideline on measles surveillance and outbreak management.

[14] Mitiku K, Bedada T, Masresha BG, Kegne W, Nafo-Traore F, Neghist Tesfaye AY (2011). Progress in measles mortality reduction in Ethiopia, 2002-2009. JID.

[15] WHO/UNICEF/FMOH (2010). Ethiopian National Expanded Programme on Immunization: Comprehensive Multi-Year Plan 2011–2015.

[16] Ethiopian Health and Nutrition Research Institute (2012). Public Health Emergency Management Guideline.

[17] Parlament FDR of E. Ethiopian Public Health Institute Establishment Council of Ministers Regulation No. 301/2013. Federal Negarit Gazette]. 2014;7175. Available from: https://chilot.me/wp-content/uploads/2017/04/regulation-no-301-2013-ethiopian-public-health-institute-establishment.pdf.

[18] World Health Organization (WHO) (2003). WHO–recommended standards for surveillance of selected vaccine-preventable diseases. WHO-Recommended Stand Surveill Sel Vaccine-Preventable Dis.

[19] World Health Organization (2010). Monitoring progress towards measles elimination. Wkly Epidemiol Rec.

[20] Central Statistical Agency. 2007 census report: Addis Ababa statistical report. Vol. 12, Central Statistical Agency. Addis Ababa, Ethiopia; 2007. Available from: http://www.csa.gov.et/index.php/census-report/complete-report/census-2007.

[21] Altizer S, Dobson A, Hosseini P, Hudson P, Pascual M (2006). REVIEWS AND seasonality and the dynamics of infectious diseases. Ecol Lett.

[22] Dollimore N, Cutts F, Binka FN, Ross DA, Morris SS, Smith PG (1997). Measles incidence, case fatality, and delayed mortality in children with or without vitamin a supplementation in rural Ghana. Am J Epidemiol.

[23] Nandy R, Handzel T, Zaneidou M, Biey J, Coddy RZ, Perry R (2006). Case-fatality rate during a measles outbreak in eastern Niger in 2003. Clin Infect Dis.

[24] Wolfson LJ, Grais RF, Luquero FJ, Birmingham ME, Strebel PM (2009). Estimates of measles case fatality ratios: a comprehensive review of community-based studies. Int J Epidemiol.

[25] Minetti A, Kagoli M, Katsulukuta A, Huerga H, Featherstone A, Chiotcha H (2013). Lessons and challenges for measles control from unexpected large outbreak. Malawi Emerg Infect Dis.

[26] Enquselassie F, Ayele W, Dejene A, Messele T, Abebe A, Cutts FT (2003). Seroepidemiology of measles in Addis Ababa, Ethiopia: implications for control through vaccination. Epidemiol Infect.

[27] Sudfeld CR, Navar AM, Halsey NA (2010). Effectiveness of measles vaccination and vitamin a treatment. Int J Epidemiol.

[28] Rosenthal SR, Clements CJ (1993). Two-dose measles vaccination schedules. Bull World Health Organ.

